# Optimization-Driven Hybrid Machine Learning Framework for Brain Tumor Classification in MRI with Metaheuristic Feature Selection

**DOI:** 10.3390/diagnostics16050819

**Published:** 2026-03-09

**Authors:** Yasin Özkan, Yusuf Bahri Özçelik, Aytaç Altan

**Affiliations:** 1 Department of Computer Technologies, Zonguldak Bülent Ecevit University, Zonguldak 67100, Türkiye; yasin.ozkan@beun.edu.tr; 2 Department of Electrical Electronics Engineering, Zonguldak Bülent Ecevit University, Zonguldak 67100, Türkiye; ybahri.ozcelik@beun.edu.tr

**Keywords:** brain tumor classification, magnetic resonance imaging, superb fairy-wren optimization, metaheuristic feature selection, hybrid machine learning framework

## Abstract

**Background/Objectives:** Brain tumors are among the most severe neurological disorders, and their variability in size, morphology, and anatomical location complicates early and accurate diagnosis. Although magnetic resonance imaging (MRI) is the most reliable non-invasive modality for tumor detection, manual interpretation remains time-consuming, subjective, and susceptible to human error. This study aims to develop an optimization-driven hybrid machine learning framework for accurate and computationally efficient automatic brain tumor classification. **Methods:** The dataset includes 834 MRI images (583-training, 123-validation, 128-independent test). Because YOLOv11 detects tumor and non-tumor regions separately, the sample size doubled during region-based analysis, and all subsequent stages were conducted at the regions of interest (ROI) level. On the independent test set, YOLOv11 achieved 98.87% mAP@50, 98.54% precision, and 98.21% recall. The proposed framework combines automated tumor localization with image standardization using Gaussian noise reduction and bilinear interpolation. From the processed MR images, 39 entropy-based features were extracted. To enhance diagnostic performance and eliminate redundant information, the superb fairy-wren optimization algorithm (SFOA) was applied for feature selection and compared with particle swarm optimization (PSO), Harris hawk optimization (HHO), and puma optimization (PO). Final classification was primarily performed using k-nearest neighbors (kNN), while support vector machines (SVM) were used for comparative evaluation. **Results:** SFOA reduced the feature dimensionality from 39 to 5 features while achieving 99.20% classification accuracy on the independent test set. In comparison, PSO selected 10 features, HHO selected 6 features and PO selected 10 features, all achieving 98.45% accuracy. The best performance obtained with SVM was 98.45% accuracy (HHO-SVM), which remained lower than the 99.20% achieved by the proposed SFOA-kNN model. **Conclusions:** The results indicate that combining entropy-based feature extraction with SFOA-driven feature selection and kNN classification significantly enhances diagnostic accuracy while reducing computational complexity, highlighting the strong potential of the proposed framework for integration into computer-aided diagnosis systems to support clinical decision-making.

## 1. Introduction

Brain tumors, characterized by the abnormal and uncontrolled proliferation of cells within the cranial cavity, remain among the most critical and life-threatening neurological disorders. Due to the brain’s central role in regulating emotional, cognitive and motor functions, the presence of tumor formations often leads to severe structural and functional impairments [1]. Depending on their histological and molecular features, brain tumors are typically classified as either benign or malignant, with each category having a profound impact on patient survival and quality of life. Among the most common tumor types are gliomas, pituitary adenomas, and meningiomas, each of which is characterized by distinct biological behaviors and therapeutic implications [2,3].

The early and accurate diagnosis of brain tumors remains a clinical priority. Delayed detection or misclassification not only limits treatment options but also decreases survival rates and worsens the overall prognosis. Manual interpretation of magnetic resonance imaging (MRI) images for diagnostic purposes is often hindered by tumor heterogeneity in terms of type, location and size, thereby increasing the likelihood of human error. Consequently, there is a growing need for computer-aided diagnosis (CAD) systems, designed to support radiologists by improving diagnostic efficiency and reliability [4,5].

Recent breakthroughs in artificial intelligence, particularly in machine learning (ML) and deep learning (DL), have transformed medical imaging. Convolutional neural networks (CNNs), due to their ability to automatically extract hierarchical features from images, have demonstrated exceptional performance in various medical imaging applications [6]. However, despite the success of single-model architectures, limitations such as overfitting, computational burden and restricted generalizability often arise. Pure deep learning-based approaches often require large-scale annotated datasets, exhibit high computational complexity, and may suffer from reduced interpretability in clinical environments. Additionally, end-to-end architectures may introduce redundant feature representations, which can negatively affect model efficiency and generalization. To address these challenges, hybrid approaches that combine the representation power of deep learning with the classification strength of traditional ML algorithms are increasingly being explored [7,8,9,10,11,12]. These frameworks offer enhanced accuracy, robustness, and adaptability, thereby holding promise for real-world clinical integration.

Despite these advancements, a clear research gap remains. Most existing studies either rely exclusively on end-to-end deep learning architectures, which may suffer from limited interpretability and computational overhead, or employ handcrafted feature extraction without systematic optimization, leading to redundant and suboptimal feature representations. The integration of real-time object localization, entropy-based feature modeling, and metaheuristic-driven feature selection within a unified hybrid framework remains limited in the current literature. This gap highlights the need for an optimization-driven diagnostic pipeline that balances localization accuracy, feature compactness, and classification robustness.

Unlike prior studies that primarily emphasize detection or segmentation accuracy, the proposed study introduces an optimization-driven hybrid diagnostic architecture that integrates YOLOv11-based localization, entropy-based feature modeling, metaheuristic feature selection, and distance-based classification within a unified pipeline, aiming to enhance diagnostic accuracy, computational efficiency, and clinical applicability. In this approach, the YOLOv11 model is first used to automatically localize tumor and non-tumor regions, thereby minimizing dependence on manual segmentation and reducing diagnostic subjectivity. The extracted regions then undergo Gaussian noise reduction and bilinear interpolation to ensure uniform image quality. Traditional handcrafted feature extraction methods, while interpretable, frequently generate high-dimensional feature spaces containing redundant and non-informative descriptors. Without effective feature selection, such redundancy may increase computational burden and reduce classifier stability. From these standardized images, 39 entropy-based features are derived to capture the underlying structural and textural characteristics of brain tissue. Entropy-based descriptors were specifically selected due to their ability to capture signal irregularity, structural complexity, and tissue heterogeneity, which are characteristic properties of tumor regions in MRI. Unlike traditional texture descriptors that primarily model spatial co-occurrence patterns, entropy measures provide a more comprehensive quantification of randomness and distributional variability within pathological tissue. To improve discriminative power while minimizing redundancy, feature selection is performed using four metaheuristic algorithms: the superb fairy-wren optimization algorithm (SFOA), which serves as the primary optimization mechanism, and particle swarm optimization (PSO), Harris hawk optimization (HHO), and puma optimization (PO), which are incorporated for comparative performance evaluation. K-nearest neighbors (kNN) is the core classification model due to its strong performance and compatibility with optimized feature subsets. Support vector machines (SVMs) are employed as secondary classifiers to examine robustness across different learning paradigms. Experimental results demonstrate that the SFOA-kNN configuration achieves the highest diagnostic performance by selecting only five highly informative features, with an accuracy rate of 99.20%. These findings underscore the effectiveness of combining Yolov11-based detection, entropy-driven feature engineering, and metaheuristic-enhanced feature selection within a hybrid ML-DL architecture and highlight the framework’s strong potential for deployment in real-time computer-aided diagnosis systems. By reducing feature dimensionality while preserving diagnostic performance, the proposed framework supports faster decision-making, lowers computational requirements, and has the potential to reduce radiologists’ workload while improving diagnostic reliability in routine clinical workflows.

The remainder of this paper is organized as follows. Section 2 provides the theoretical background of the study and presents a comprehensive review of recent developments in MRI-based brain tumor detection, with a particular emphasis on hybrid learning strategies and optimization-enhanced feature selection. Section 3 describes the proposed methodology in detail, including the Yolov11-based tumor localization framework, the pre-processing procedures, entropy-based feature extraction, and the integration of the SFOA with the kNN classifier, which forms the core of the proposed hybrid model. Section 4 presents the experimental results along with an in-depth discussion, highlighting the diagnostic performance of the proposed SFOA-kNN model and comparing it with alternative approaches. Section 5 concludes the paper by summarizing the key findings and offering insights into potential future extensions aimed at enhancing model generalizability and clinical applicability.

## 2. Background and Related Works

The classification of brain tumors using MRI has become a central research topic in medical image analysis due to the increasing clinical demand for fast, reliable and objective diagnostic support systems. Although MRI is widely recognized for its superior soft-tissue contrast and non-invasive nature, the manual examination of MRI scans remains challenging due to the substantial variation in tumor morphology across patients in terms of shape, size, intensity, texture and anatomical location. These complexities have motivated extensive research into automated diagnostic frameworks driven by artificial intelligence (AI), which are capable of improving diagnostic accuracy, reducing human error, and supporting clinical decision-making.

### 2.1. Deep Learning Approaches for Brain Tumor Classification

Deep learning, particularly CNNs, has dominated recent literature because of its ability to automatically extract hierarchical and discriminative features from MRI data. Numerous studies have established CNN-based models as highly effective tools for multi-class tumor classification tasks. For instance, Kumar et al. achieved over 97% accuracy using a ResNet-based architecture enhanced with global average pooling, demonstrating that residual connections effectively mitigate gradient degradation issues in deep models [13]. Similarly, Srinivas et al. developed the BMRI-Net architecture that incorporates the innovative PFpM activation function, outperforming traditional activations such as ReLU, Mish, and GELU, and achieving up to 99.57% accuracy on benchmark datasets [14]. Transfer learning–based models, including AlexNet, VGG16, U-Net, and improved ResNet variants, have also shown remarkable performance, with accuracy levels frequently exceeding 98–99% [15].

Other studies have investigated more advanced architectures such as DenseNet, EfficientNet, and Swin Transformer. Özkaraca et al. demonstrated that densely connected CNN architectures can achieve competitive performance under extensive cross-validation [16], while Nayak et al. reported high performance using a dense EfficientNet model with four-class classification [17]. Transformer-based designs have recently shown strong generalizability, as evidenced by Elhadidy et al., who achieved accuracies above 98% using Swin Transformer and EfficientNet hybrids [18].

### 2.2. Optimization-Enhanced Deep Learning Frameworks

Despite their success, deep learning models have limitations. They often involve high-dimensional feature representations and redundant information, as well as a significant computational burden. This makes optimization a vital component of model refinement. To address these issues, researchers have introduced metaheuristic optimization techniques to improve hyperparameter tuning, feature reduction and residual network optimization.

Mehnatkesh et al. incorporated an improved ant colony optimization (IACO) mechanism into a ResNet architecture (IACO-ResNet), achieving an accuracy of 98.69% [19]. Emam et al. optimized the ResNet50 hyperparameters using the improved hunger games search (I-HGS) algorithm and reached accuracies exceeding 99% [20]. Abd El Kader et al. proposed a differential deep CNN enriched with differential operators, achieving 99.25% accuracy on large tumor datasets [21]. These studies demonstrate the growing importance of optimization strategies in enhancing the accuracy and computational efficiency of deep learning models.

### 2.3. Hybrid Deep Learning-Machine Learning Models

A parallel trend in the recent literature is the integration of hybrid approaches that combine CNN-based feature extraction with ML classifiers. This strategy capitalizes on the rich representational capacity of deep networks, as well as the robustness, interpretability and efficiency of traditional ML algorithms.

Hybrid ensemble frameworks, such as majority voting across multiple CNN architectures (e.g., GoogleNet, AlexNet, ShuffleNet, SqueezeNet and NASNet-Mobile), have yielded excellent results, achieving accuracies above 99% [22]. Similarly, Kibriya et al. combined deep feature fusion with ML classifiers such as kNN and SVM, obtaining 99.7% accuracy on a large MRI dataset [23]. In a related hybrid approach, Sharif et al. extracted deep features using a fine-tuned DenseNet201 model and enhanced them through entropy–kurtosis-based selection and a modified genetic algorithm, followed by classification using a multiclass SVM, achieving over 95% accuracy [24]. Rinesh et al. used a hybrid pipeline incorporating k-means clustering, kNN, and firefly optimization for hyperspectral brain tumor localization, achieving robust performance [8]. Furthermore, Başaran advanced hybrid modeling by integrating handcrafted texture descriptors, specifically the gray level co-occurrence matrix (GLCM) and local binary pattern (LBP) features, with deep representations extracted from CNN architectures. These combined features were further refined through metaheuristic optimization using genetic algorithms (GA), PSO, and artificial bee colony (ABC), ultimately achieving a classification accuracy of 98.22% [11].

Recent studies continue to explore optimization-enhanced hybrid models. Semwal et al. introduced a PSO-optimized CNN–SVM framework for multiclass brain tumor classification, achieving 84.77% accuracy [25]. Likewise, Sharif et al. proposed M3BTCNet, a multimodal deep learning model incorporating differential evolution and moth flame optimization, reporting up to 99.06% accuracy on the BRATS dataset [26]. While these approaches demonstrate the effectiveness of metaheuristic-driven deep feature refinement, they primarily emphasize hyperparameter tuning or segmentation-oriented classification rather than compact feature-space optimization within a unified detection–classification pipeline.

### 2.4. Research Gaps and Motivations

Although the literature shows significant progress, there are still several critical gaps:

• Most CNN-based studies rely solely on end-to-end deep learning, which often results in high dimensionality, redundant features and increased computational costs.
• Although hybrid frameworks exist, they frequently lack an optimization-driven mechanism to systematically reduce feature redundancy while preserving discriminative information.
• Feature selection is often neglected or handled sub-optimally, leading to unnecessary complexity and reduced generalizability.
• The dominance of DL classifiers means there has been limited exploration of efficient, lightweight ML classifiers (e.g., kNN) integrated with optimized feature subsets.
• There is a lack of comparative studies involving multiple metaheuristic algorithms, particularly in the context of MRI-based tumor classification.

These gaps highlight the need for a lightweight, optimization-enhanced hybrid diagnostic framework that strikes a balance between accuracy, interpretability and computational efficiency.

### 2.5. Contribution of the Proposed Method

Inspired by these observations, the present study introduces a hybrid framework that overcomes the limitations of previous research:

• Yolov11-based tumor localization reduces manual intervention and ensures robust region extraction.
• Entropy-based feature engineering captures meaningful structural and textural information from MRI scans.
• SFOA is introduced as the primary feature selection mechanism to reduce feature dimensionality and maximize discriminative power.
• kNN is adopted as the main classifier, offering computational simplicity, high interpretability, and strong performance when paired with optimized feature subsets.
• PSO, HHO, PO and SVM are used solely for comparative evaluation, enabling a rigorous benchmarking against existing optimization and classification strategies.

This design aims to provide a computationally efficient, highly accurate and clinically applicable approach to MRI-based brain tumor classification, addressing the limitations identified in the existing literature.

## 3. Material and Methods

This section presents the proposed optimization-driven hybrid framework for MRI-based brain tumor classification. The methodology is designed as a modular yet tightly integrated pipeline that combines automated region localization, preprocessing, entropy-based feature extraction, metaheuristic-driven feature selection, and machine learning-based classification. The overall aim is to achieve high diagnostic accuracy while maintaining low computational complexity and strong interpretability, thereby ensuring suitability for real-world clinical deployment.

### 3.1. YOLOv11-Based Tumor Localization

YOLOv11 is one of the latest real-time object detection models developed by Ultralytics, building upon previous YOLO architectures, particularly YOLOv8 [27]. Its architectural design introduces several significant enhancements, most notably the C3k2 blocks, the spatial pyramid pooling–fast (SPPF) layer, and the cross-stage partial with parallel spatial attention (C2PSA) mechanism. These components collectively enable a balanced trade-off between parameter efficiency and detection accuracy, making YOLOv11 particularly suitable for medical imaging applications where both precision and computational cost are critical [27,28,29].

The overall YOLOv11 architecture follows the classical YOLO paradigm, consisting of three main components: backbone, neck, and head. The backbone is responsible for extracting multi-scale feature maps from the input MRI images, capturing both low-level texture details and high-level semantic information. The neck aggregates these features across different spatial resolutions, improving robustness against variations in tumor size and shape. Finally, the head produces the bounding box coordinates and class probabilities for tumor and non-tumor regions.

Fundamentally, all components of the YOLOv11 architecture rely on two-dimensional convolution operations as their primary computational building block. Therefore, to interpret the internal operations of the backbone, neck, and head modules, it is essential to understand the mathematical formulation of 2D convolution. Let the input feature map be denoted by $X\in R^{C_{in}\times H\times W}$.

For a convolution operation using a k×k kernel, the output feature map at channel c, spatial location (i,j), is mathematically expressed in:

(1) $$Y_{c}\left(i,j\right)=\sum_{m=1}^{C_{in}}\sum_{u=1}^{k}\sum_{v=1}^{k}W_{c,m}\left(u,v\right)X_{m}\left(i+u,j+v\right)+b_{c}$$

where $W_{c,m}$ represents the convolution kernels and $b_{c}$ denotes the bias term. This formulation constitutes the core operation applied across standard convolution layers, as well as within advanced architectural modules such as C3k2 and C2PSA [27].

The deep feature maps generated by the YOLOv11 backbone are subsequently processed by the SPPF block to enhance multi-scale contextual representation. SPPF is an accelerated variant of the traditional Spatial Pyramid Pooling structure, designed to integrate multiple max-pooling operations with different kernel sizes into a single module, thereby reducing computational cost while strengthening multi-scale information flow [30]. For an input feature map X, the three parallel pooling operations are mathematically defined in

(2) $${SPPF}_{i}(X)=MaxPool(X,k_{i})$$

where $k_{1}<k_{2}<k_{3}$ denote the pooling kernel sizes. The final SPPF output, obtained by concatenating these pooled features along the channel dimension, is expressed in

(3) $${SPPF}_{out}=Concat({SPPF}_{1},\;{SPPF}_{2},\;{SPPF}_{3})$$

This design allows features corresponding to different receptive field sizes to be jointly represented, improving detection performance for both small and large structures. Following SPPF, the C3k2 block is employed as a refined feature processing module. C3k2 replaces the C2f structure used in YOLOv8 and represents a more efficient variant of the Cross-Stage Partial (CSP) scheme [27]. In this design, part of the input is propagated directly, while the remaining portion is processed through two sequential small-kernel convolutions. The parameter count of a standard convolution layer and that of the C3k2 structure are mathematically described in

(4) $$P_{std}=C_{in}\times C_{out}\times k\times k$$

(5) $$P_{C3k2}=C_{in}\times C_{mid}\times 3\times 3+C_{mid}\times C_{out}\times 3\times 3$$

with $C_{mid}<C_{out}$. This configuration reduces the overall parameter and FLOP cost while preserving an effective receptive field similar to larger convolutions.

**Figure 1:**
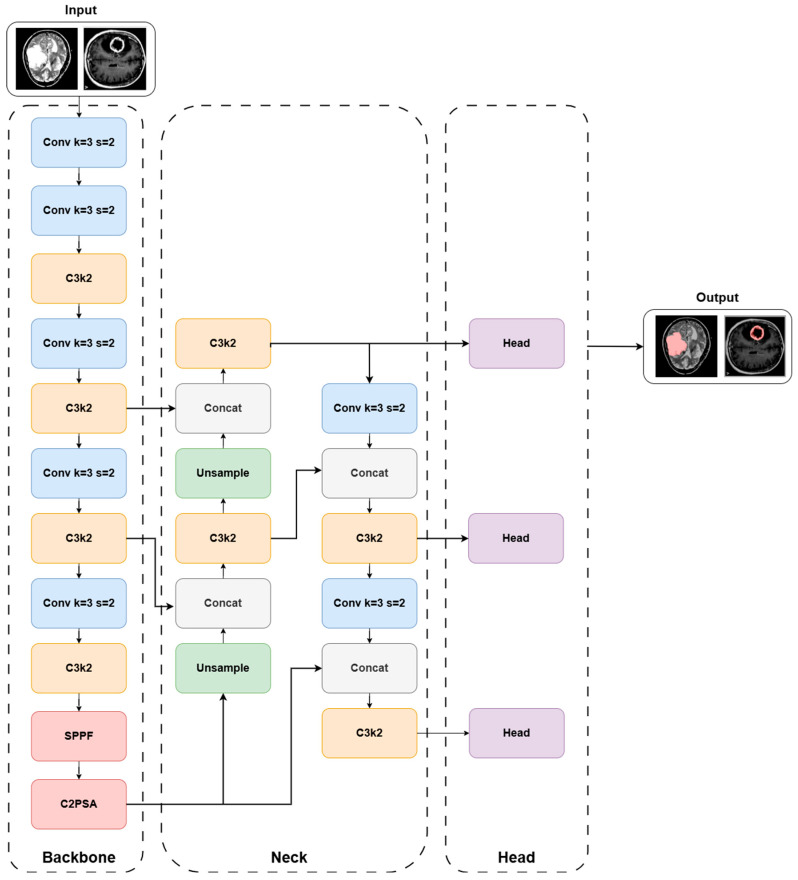
Diagram of the YOLOv11 architecture.

Another key component integrated into the neck stage is the C2PSA. Empirically, the use of C3k2 contributes to approximately 22% fewer parameters in YOLOv11m compared to YOLOv8m while achieving higher mAP50–95 values on the COCO dataset [27] module. This module combines the CSP framework with a Pyramid Squeeze Attention (PSA)-based attention mechanism, enabling selective emphasis of both spatial and channel-wise information. For an input feature map $U\in R^{C\times H\times W}$, the multi-scale feature extraction and attention-based rescaling operations are mathematically expressed in

(6) $$F=\sum_{s=1}^{S}\emptyset_{s}(U)$$

(7) $$U^{\prime }=\sigma (F)⊙U$$

where $\phi_{s}(\cdot )$ denotes multi-scale feature extraction operators, σ(⋅) is the attention scaling function, and ⊙ represents element-wise multiplication. The overall YOLOv11 architecture employed in this study is illustrated in Figure 1.

In the proposed framework, YOLOv11 is used to automatically detect both tumor and non-tumor regions in MRI images. The predicted bounding boxes are cropped to generate regions of interest (ROIs), which are subsequently forwarded to the feature extraction stage.

To ensure reproducibility, the YOLOv11 model was trained with explicitly defined hyperparameters. The training process was conducted for 100 epochs with a batch size of 16 using the Adam optimizer. The initial learning rate was set to 0.001, and a cosine learning rate scheduling strategy was applied to gradually decrease the learning rate throughout the training process. In addition, weight decay was set to 5 × 10^−4^ to reduce overfitting risk. Early stopping with a patience value of 15 epochs was employed based on validation loss to prevent unnecessary training once convergence was achieved.

### 3.2. Feature Extraction

Following the automated localization of tumor and non-tumor regions using YOLOv11, the next critical stage of the proposed framework focuses on feature extraction. The purpose of this stage is to transform the visual and structural information contained within the localized ROIs into a compact and discriminative numerical representation suitable for machine learning–based classification. Consistent with the motivation outlined in Section 1 and Section 2, the feature extraction strategy emphasizes robustness, interpretability, and sensitivity to subtle structural variations in brain tissue.

In the proposed approach, each ROI extracted from the MRI images is first converted from a two-dimensional spatial representation into a one-dimensional signal. This transformation is performed by reorganizing pixel intensity values into a sequential form, enabling the use of signal-based complexity measures while preserving essential statistical and structural characteristics of the tissue. The one-dimensional representation significantly reduces computational burden and allows efficient extraction of nonlinear descriptors that are difficult to capture using conventional spatial features alone.

Entropy-based feature extraction is then applied to these one-dimensional signals. Entropy measures are particularly well suited to the analysis of biomedical images and signals, as they quantify uncertainty, irregularity and complexity—properties that are strongly associated with pathological tissue behavior. Tumor regions typically exhibit higher structural irregularity and more heterogeneous intensity distributions than healthy tissue, meaning that entropy-based descriptors are highly effective for tumor classification tasks.

In this study, a comprehensive set of 39 entropy-based features is extracted from each transformed signal. The feature set includes diverse entropy formulations designed to capture both local and global characteristics of the signal. These encompass classical entropy measures as well as advanced nonlinear descriptors that model signal unpredictability, variability, and distributional complexity. In addition to single-signal entropy measures, cross-entropy-based features are also considered to characterize interactions and dependencies between tumor and non-tumor signal representations.

The extracted entropy features collectively form a high-dimensional feature space that encodes multiple aspects of tissue complexity. While this rich representation enhances discriminative capability, it also introduces potential redundancy and correlation among features. Consequently, directly using the complete feature set for classification may increase computational complexity and reduce generalization performance. This observation motivates the subsequent feature selection stage, where a metaheuristic optimization algorithm is employed to identify the most informative and compact subset of features without compromising diagnostic accuracy.

Overall, the entropy-based feature extraction stage serves as a crucial bridge between deep learning-based region localization and machine learning-based classification. By translating localized MRI data into a structured and interpretable numerical form, this stage enables the proposed framework to leverage both the representational power of deep models and the efficiency of optimized machine learning classifiers.

### 3.3. Superb Fairy-Wren Optimization Algorithm

The superb fairy-wren (*Malurus cyaneus*) has been extensively investigated in ecology due to its adaptive foraging strategies, complex acoustic communication, and evasive maneuvers under predation pressure. This rich behavioral repertoire provides a natural modeling foundation for population-based heuristic algorithms, as it enables a dynamic balance between large-scale resource exploration and local convergence. Inspired by these observations, the SFOA was developed as a metaheuristic method that adaptively regulates the exploration–exploitation trade-off [31,32].

In SFOA, each candidate solution represents an individual superb fairy-wren, and the position of the i-th individual in a D-dimensional search space is defined as

(8) $$X_{i}=\left[x_{i1},x_{i2},\ldots ,x_{iD}\right],\;\;\;i=1,\ldots ,N$$

The SFOA optimization cycle is structured around three main phases inspired by the biological behaviors of superb fairy-wrens: (i) the Young Birds Growth Stage, which enhances exploration by mimicking the rapid learning and experience acquisition of juveniles; (ii) the Breeding and Feeding Stage, which models cooperative breeding and caregiving in order to intensify local convergence around the best solution; and (iii) the Avoiding Natural Enemies Stage, which simulates evasive maneuvers under predation pressure in order to increase population diversity and facilitate escape from local minima. Together, these components ensure a balanced and adaptive search process.

#### 3.3.1. Young Birds Growth Stage

The first phase models the rapid growth and experience acquisition of juvenile superb fairy-wrens. In nature, young individuals learn to explore food resources more effectively in a short time [31]. This behavior is translated into a wide-jump update operator in SFOA to strengthen global exploration, as formulated in

(9) $$X_{i}^{new}=X_{i,j}^{t}+rand\times \left(X_{i,j}^{t}-X_{i,j}^{t-1}\right)$$

Here, rand∈[0,1] is a random coefficient that scales the directional difference between the current position and the previous-iteration position $\left(X_{i,j\;}^{t}-\;X_{i,j}^{t-1}\right)$. This difference vector mathematically represents the irregular yet purposeful movements observed during juvenile learning. The operator enables individuals to perform large jumps in the search space, preserving diversity and substantially reducing the risk of premature convergence to local minima. Therefore, the growth stage constitutes one of the key mechanisms that determines the global exploration capability of SFOA.

#### 3.3.2. Breeding and Feeding Stage

The second phase is based on the cooperative breeding behavior of superb fairy-wrens, where incubation and offspring care are carried out with contributions from multiple individuals. In SFOA, this biological mechanism is mapped into an exploitation operator that generates intensified local search. The best individual of the population, $X_{b}$, is used as a central guiding position, and the update rule is given in

(10) $$X_{i}^{new}=X_{b,j}+C\times p\times \left(X_{i,j}^{t}-X_{b,j}\right)$$

In this equation, C=0.8 is a constant control coefficient that regulates the tendency of individuals to move toward the best solution and is kept unchanged throughout all iterations, ensuring a balanced convergence pattern in the breeding–feeding stage. The factor p represents the maturity level of the algorithm and is defined as the ratio of the current number of evaluations to the maximum evaluation budget, as expressed in

(11) $$p=\frac{{FE}_{s}}{{MaxFE}_{S}}$$

As iterations proceed, p increases and strengthens the movement around the best solution, enabling broader search in early stages and more focused convergence in later stages. This mechanism causes individuals to increasingly concentrate around the region of the optimal solution as the process progresses. While early-stage convergences remain limited, later iterations exhibit stronger movements that guide individuals more decisively towards the optimum. Therefore, the algorithm initially explores widely and then performs deeper, more focused convergence, progressing towards the optimum in a controlled manner.

#### 3.3.3. Avoiding Natural Enemies Stage

This phase models abrupt directional changes, alarm calls and complex escape maneuvers executed under predation pressure. The same process is used in the algorithm as an escape-and-reexploration mechanism to move away from local minima. The mathematical formulation is provided in

(12) $$X_{i,j}^{new}=X_{i,j}^{t}+l\times k\times \left(X_{b,j}-X_{i,j}^{t}\right)$$

where l denotes a random step size generated based on a Lévy flight, this enables large jumps in the search space, allowing the algorithm to move in unexpected directions and avoid local minima. The other component, k, is an adaptive flight coefficient that is updated according to the alarm frequency during the iterative process. This coefficient dynamically adjusts flight behavior, determining both movement intensity and the sensitivity of avoidance maneuvers. Its formulation is given in Equations (13) and (14). These two parameters work together to make the escape mechanism more flexible and effective.

(13) $$k=0.2\times sin\;\;\left(\frac{\pi }{2}-\omega \right)\;\;$$

(14) $$\omega =\frac{\pi }{2}\times \frac{{FE}_{s}}{{MaxFE}_{S}}$$

This operator has two major advantages. Firstly, the Lévy flight component improves global exploration by enabling large jumps across the search space. Secondly, the adaptive sine-based coefficient increases stability by making movements more controlled as iterations progress. Together, they are particularly beneficial in solving multimodal problems, facilitating effective escape from local traps and enabling broader coverage of the search space, thereby strengthening overall performance.

#### 3.3.4. Implementation of SFOA

Implementing SFOA begins with defining key parameters, such as the population size (N), the problem dimension (D) and the maximum number of evaluations (${MaxFE}_{S}$). Initially, individuals are randomly distributed across the search space to ensure diversity. In each iteration, one of three behavioral mechanisms is applied to update positions: the exploration phase (juvenile learning), the local convergence phase (cooperative breeding) and the diversity-increasing escape phase (predator avoidance). After each update, the new position is evaluated using the objective function, and the update is accepted if improvement is achieved. This process continues until the evaluation budget is exhausted, ensuring a broad search in the early iterations and a greater concentration around the best solution in the later ones [33].

The overall update mechanism that unifies the three behavioral stages is given in Equation (15), representing the position-update logic of SFOA.

(15) $$X_{i}^{t+1}=\left\{\begin{array}{c} X_{i}^{t}+rand\times \left(X_{i}^{t}-X_{i}^{t-1}\right),\;\;\;\;\;\;\;\;\;\;\;\;\;\;\;\;\;\;\;\;\;\;\;\;\;\;\;\;\;\;\;\;\;Growth\;Stage\;\;\;\;\;\;\;\;\;\;\;\;\;\;\;\;\;\;\\ X_{b}+C\times p\times \left(X_{i}^{t}{-X}_{b}\right)\;,\;\;\;\;\;\;\;\;\;\;\;\;\;\;\;\;\;\;\;\;\;\;\;\;Breeding\;and\;Feeding\;Stage\;\;\;\;\\ X_{i}^{t}+l\times k\times \left(X_{b}-X_{i}^{t}\right)\;\;\;\;\;\;\;\;\;\;\;\;\;\;\;\;\;\;\;\;\;\;\;\;\;\;\;\;Avoiding\;Enemies\;Stage\;\;\;\;\;\;\;\;\;\;\;\; \end{array}\right.$$

In this study, SFOA is used to select the most effective and discriminative features for classification from a high-dimensional, entropy-based set of features. The features extracted from tumor and non-tumor regions, detected by YOLOv11 and converted into one-dimensional form, are optimized by SFOA before being passed to the classification stage. This eliminates unnecessary, noisy or low-contribution components. This process reduces computational cost and substantially improves the classifier’s overall performance. Consequently, SFOA acts as a vital optimization layer that enhances the performance of the proposed hybrid architecture.

### 3.4. k-Nearest Neighbors (kNN) Classifier

The kNN classifier is a fundamental machine learning method based on instance-based learning and the lazy learning paradigm [34]. Unlike parametric models, which learn an explicit functional mapping during training, kNN stores training instances and only performs classification at inference time. Despite its conceptual simplicity, kNN is widely regarded as a powerful classifier because it effectively exploits local data characteristics and decision structures inherent in the feature space [35].

The operating principle of kNN relies on identifying the closest neighbors of a query sample within the training set. When presented with a new instance, the algorithm computes the distances to all the training samples using the selected distance metric, and then determines the class label via majority voting among the k nearest neighbors [35]. The choice of distance function (e.g., Euclidean, Manhattan or Minkowski) and the selection of k are two key factors that directly influence classification performance. Smaller k values may lead to sharper decision boundaries but can increase sensitivity to noise, whereas larger k values tend to produce smoother and more generalizable boundaries [36].

Thanks to its ability to represent non-linear decision boundaries and its flexibility in distance-based modeling, kNN has been successfully applied to image classification, text mining, pattern recognition, and medical diagnosis tasks [37,38,39,40]. In the proposed framework, kNN is adopted as the primary classifier because the features localized by YOLOv11 and optimized by SFOA can be effectively separated through similarity-driven decision rules. As a non-parametric learner, kNN rapidly adapts to the statistical structure of entropy-based descriptors and provides reliable discrimination between tumor and non-tumor samples based on proximity in the optimized feature space.

### 3.5. Performance Metrics for Classification

In this study, entropy-based features are extracted from tumor and non-tumor regions obtained from MRI brain scans. The most informative subset is then selected using SFOA, and classification is performed using the kNN classifier. To evaluate the classification performance, the confusion-matrix components True Positive (TP), True Negative (TN), False Positive (FP), and False Negative (FN) are used. TP denotes the number of tumor samples correctly classified as tumor, whereas TN represents the number of non-tumor samples correctly classified as non-tumor. Conversely, FP corresponds to non-tumor samples that are incorrectly labeled as tumor, and FN indicates tumor samples that are incorrectly labeled as non-tumor. Based on these quantities, accuracy, precision, recall, and F1-score are computed; recall is particularly critical in medical classification tasks because it reflects how many true tumor cases are correctly detected, while the F1-score provides a balanced measure under potential class imbalance. The mathematical definitions of these metrics are given in Equations (16)–(19).

(16) $$Accuracy=\frac{TP+TN}{TP+TN+FP+FN}$$

(17) $$Precision=\frac{TP}{TP+FP}$$

(18) $$Recall=\frac{TP}{TP+FN}$$

(19) $$F1\text{-}\mathrm{score}=2\times \frac{Precision\times Recall}{Precision+Recall}$$

### 3.6. Framework of the Proposed Brain Tumor Classification Model

**Figure 2:**
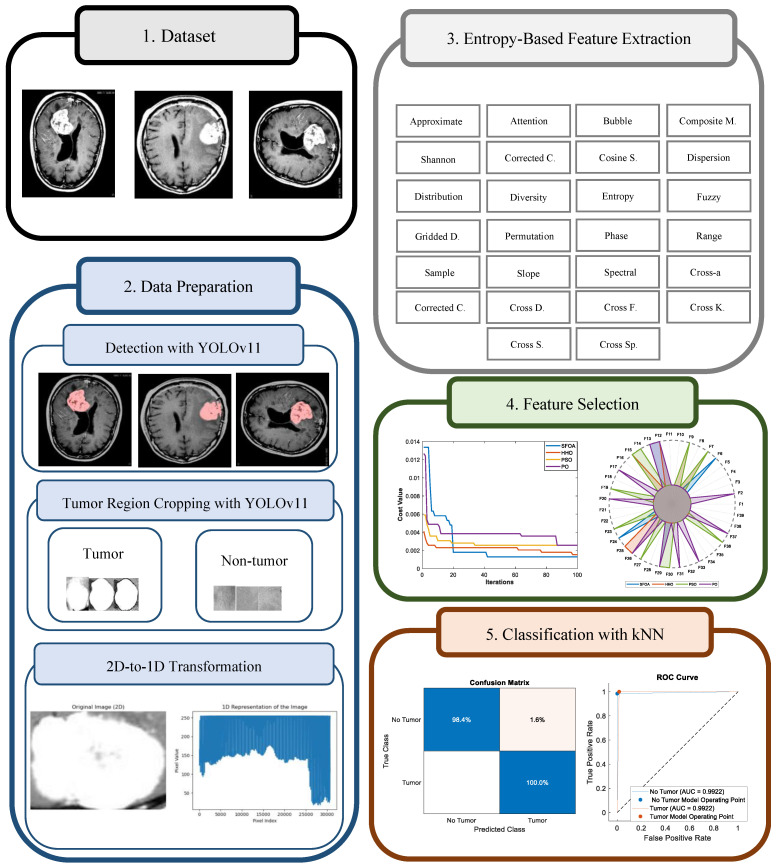
Framework of the proposed hybrid pipeline for brain tumor classification.

The proposed hybrid brain tumor classification framework is shown in Figure 2. The processing steps of the model are summarized below.

Step 1: Automatic detection of tumor and non-tumor regions (YOLOv11)

In the first step, tumor and non-tumor regions in brain MRI images are automatically detected using the YOLOv11 object detection model. YOLOv11 identifies abnormal structures in the images with high accuracy and produces bounding boxes that indicate the spatial locations of tumor-containing and tumor-free regions. These localization results are used as input for the subsequent stages.

Step 2: Extraction of tumor and non-tumor regions and 1D transformation

Using the bounding boxes generated by YOLOv11, both tumor and non-tumor regions are accurately cropped from the MRI images. Each cropped region is obtained as an independent sample. To obtain a consistent and processable data structure for classification, these two-dimensional image regions are converted into one-dimensional representations. This transformation preserves tumor morphology and healthy tissue characteristics while enabling efficient numerical processing.

Step 3: Entropy-based feature extraction

A total of 39 entropy-based features are extracted from the one-dimensional representations. These features quantitatively describe textural diversity and statistical distributions within the regions. The extracted entropy features are designed to enhance discrimination between tumor tissue and healthy brain tissue.

Step 4: Feature selection using SFOA and metaheuristic comparison

The most discriminative subset of the extracted features is identified by performing feature selection using the SFOA. The goal of this step is to remove redundant and less informative features while preserving classification performance. The same feature selection process is also applied using PSO, HHO, and PO, allowing for a metaheuristic comparison.

Step 5: Classification stage (kNN and SVM)

In the final step, the optimized feature subset is provided as input to the kNN classifier, which is used as the primary classification algorithm. SVMs are also applied to provide a comparative evaluation and analyze classification performance under different learning strategies.

In summary, the proposed framework establishes a comprehensive, fully automated pipeline that integrates region localization based on YOLOv11, entropy-based feature extraction, metaheuristic-driven feature selection, and machine learning classification. Each methodological component is designed to complement the others, ensuring both discriminative power and computational efficiency. The following section builds upon this methodological foundation by quantitatively evaluating the performance of the proposed approach. Section 4 presents comprehensive experimental results, including feature selection behavior, classification accuracy, and comparative analyses with alternative optimization and classification strategies, and discusses how each stage of the proposed framework contributes to the overall diagnostic performance.

## 4. Results and Discussion

This section presents a comprehensive experimental evaluation of the proposed hybrid brain tumor classification framework. First, the dataset characteristics and experimental setup are described to provide context for reproducibility and fair assessment. Next, the effectiveness of the feature selection process using the SFOA-kNN configuration is analyzed in terms of convergence behavior, selected feature subsets, and computational efficiency. Finally, the classification performance is evaluated and discussed through quantitative metrics and comparative analyses, highlighting the contribution of each stage of the framework to diagnostic accuracy and robustness.

### 4.1. Dataset

The experimental evaluation was conducted using an open-access Brain Tumor MRI dataset provided by IOTSEECS through the Roboflow Universe platform [41]. The dataset is publicly available and offers standardized MRI images to facilitate reproducible research. All images are provided at a uniform spatial resolution of 640×640 pixels, ensuring consistency in preprocessing, region extraction, and feature computation across all samples.

**Figure 3:**
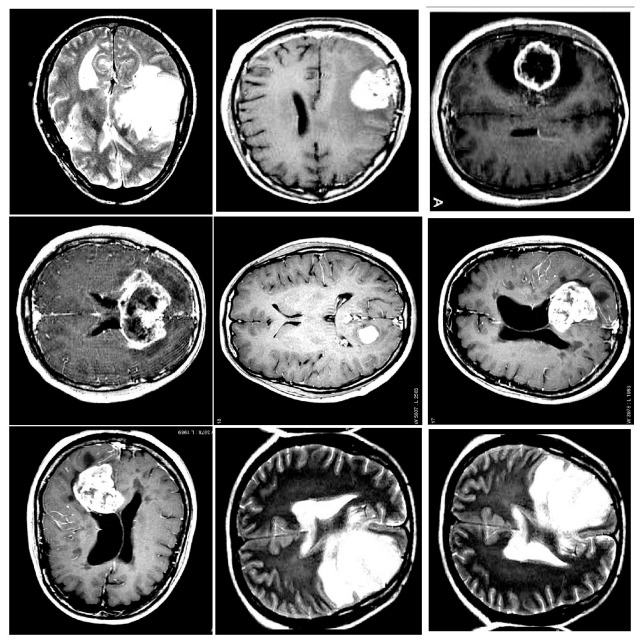
Dataset structure and sample distribution used in this study.

The dataset consists of a total of 834 MRI images, including both tumor and non-tumor cases, which supports a binary classification setting aligned with the objective of this study. To ensure reliable model training and unbiased performance evaluation, the dataset was divided into three mutually exclusive subsets: 583 images for training, 123 images for validation, and 128 images for testing. YOLOv11 was used to detect tumor regions in MRI images containing tumors, as well as to identify normal brain regions in MRI images that did not include tumors. Based on these detections, a region-based representation was constructed by extracting ROIs from the annotated areas. These ROIs correspond to localized image patches that capture either pathological tissue characteristics or normal brain structures. In this context, tumorous ROIs were obtained from MRI images containing tumors, while non-tumorous ROIs were extracted from images without tumor findings. This design ensures that tumorous and non-tumorous samples originate from independent MRI images, thereby preventing any potential pathological cross-contamination between classes. Moreover, since dataset partitioning was conducted prior to ROI extraction, the proposed data preparation strategy effectively eliminates the risk of data leakage and guarantees that no ROI derived from the same MRI image appears in different subsets. A notable characteristic of the dataset is the considerable variability in tumor size, shape, and visual appearance, reflecting realistic clinical heterogeneity. This diversity introduces additional complexity to automated detection and classification tasks and therefore provides a suitable benchmark for evaluating the robustness and generalization capability of the proposed hybrid framework. Figure 3 provides an overview of the dataset composition and subset distribution, summarizing the data used in the experimental analysis.

All experiments were conducted in a controlled and standardized computational environment using MATLAB 2022b. The experimental platform comprised a research-grade workstation equipped with an Intel Core i7-12700H processor, 16 GB of system memory, and a 6 GB VRAM NVIDIA RTX 3060 graphics processing unit. This hardware configuration is representative of a realistic and widely available setup in contemporary academic research laboratories. Using a consistent hardware and software environment for all experiments enables different optimization and classification strategies to be compared fairly, thereby enhancing the reproducibility and reliability of the reported results.

### 4.2. YOLOv11 Detection Performance Analysis

Before proceeding to feature extraction and classification, the detection capability of the YOLOv11 model was quantitatively evaluated to ensure the reliability of the ROI generation stage. Accurate tumor localization is critical, as all subsequent entropy-based feature computations depend directly on the detected regions.

Detection performance was assessed on the independent test subset using standard object detection metrics, including mean Average Precision at IoU threshold 0.5 (mAP@50), precision, and recall. The YOLOv11 model achieved a mAP@50 of 98.87%, with a precision of 98.54% and recall of 98.21%. These results indicate high localization accuracy and strong sensitivity in identifying tumor regions within MRI images.

In this study, tumor regions were defined based on the bounding box annotations provided in the dataset. Following detection, the predicted bounding box area was cropped and treated as the tumor ROI. Non-tumor regions were defined as the remaining brain tissue areas outside the detected tumor bounding boxes within the same MRI image. This approach ensures spatial separation between pathological and healthy tissue regions prior to entropy-based feature extraction.

Since the detection model was trained specifically to identify tumor instances, any false positive detection directly corresponds to misclassification of healthy tissue. The high precision value indicates a low false positive rate, confirming reliable discrimination between tumor and non-tumor regions at the detection stage. Similarly, the high recall value demonstrates that the majority of annotated tumor regions were successfully localized. Overall, the strong detection performance validates the robustness of the ROI extraction process and ensures that the subsequent SFOA-based feature optimization and classification stages operate on accurately localized anatomical structures.

In addition to the overall detection performance, the capability of YOLOv11 to handle small tumor instances is supported by its multi-scale feature extraction architecture. The backbone–neck structure integrates feature pyramid representations, allowing information from different spatial resolutions to be aggregated. The SPPF module enhances contextual awareness, while the C2PSA attention mechanism strengthens spatial sensitivity to fine-grained structures.

### 4.3. Feature Selection with SFOA-kNN

This subsection presents a detailed analysis of the feature selection stage based, which is based on the SFOA combined with a kNN classifier. The objective of this stage is not only to reduce the dimensionality of the extracted feature space but also to identify the most discriminative entropy-based features that contribute to reliable tumor classification.

Starting from the original set of 39 entropy-based features, the feature selection process is formulated as a wrapper-based optimization problem. In this formulation, each candidate solution generated by the SFOA corresponds to a binary feature selection vector, where selected and excluded features are encoded as follows:

(20) $$X_{i}=\left[x_{i1},x_{i2},\ldots ,x_{iD}\right],\;\;\;\;x_{ij}\in \left\{0,\;1\right\},\;D=39$$

The candidate solutions evolve according to the SFOA update mechanisms defined in Equations (8)–(15), which model behaviors such as exploration, exploitation and escape. The classification performance of each candidate feature subset is evaluated using the kNN classifier, and optimization is guided by a

(21) $$Fitness=\alpha \times (1-Accuracy)+(1-\alpha )\times \left(\frac{N_{s}}{\;N_{t}}\right)$$

where Accuracy denotes the kNN classification accuracy on the validation set, $N_{s}$ is the number of selected features, $N_{t}$ is the total number of extracted features (39), and α controls the trade-off between maximizing accuracy and minimizing feature dimensionality.

The parameter settings used for SFOA, PSO, HHO, and PO are summarized in Table 1. All metaheuristic algorithms were executed under identical stopping criteria and evaluation budgets to ensure a fair and unbiased comparison. By explicitly reporting these parameters, Table 1 provides transparency and reproducibility, which are essential requirements for rigorous experimental evaluation in medical image analysis.

The wrapper-based optimization approaches were applied to entropy-based feature representations generated for brain tumor classification. Table 2 reports the total computational runtimes required during the feature selection phase and highlights notable differences in computational cost among the employed optimization algorithms. The quantitative results clearly demonstrate that the SFOA achieved the shortest execution time. These results suggest that SFOA offers a computationally efficient optimization mechanism, particularly in high-dimensional entropy-based feature spaces, and demonstrates favorable scalability characteristics. This efficiency advantage is particularly important for clinical decision-support systems, where computational overhead must be minimized.

By contrast, the longer execution times observed for the HHO, PSO, and PO algorithms suggest that these approaches become more computationally demanding as the search space expands, resulting in increased processing complexity and time consumption. Overall, the results in Table 2 suggest that SFOA offers a more balanced solution, not only in terms of feature selection effectiveness but also with respect to computational efficiency and time complexity.

**Figure 4:**
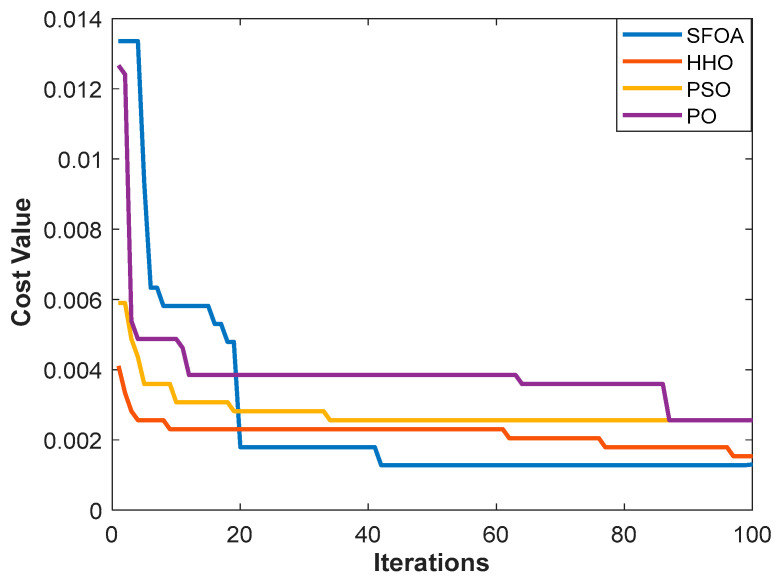
Convergence behavior of the feature selection algorithms across iterations.

The convergence behaviors of the SFOA, HHO, PSO, and PO algorithms during the feature subset selection process are illustrated in Figure 4. As can be seen, SFOA shows a rapid improvement trend from the initial iterations and achieves a highly stable convergence pattern after around 40 iterations. This behavior suggests that SFOA establishes an effective exploration mechanism at an early stage of the optimization process, demonstrating robust resistance to premature convergence towards local optima. Conversely, HHO, PSO, and PO exhibit more gradual convergence characteristics, producing incremental improvements in the objective function until the later iterations. While HHO shows a relatively stable convergence pattern, PSO and PO exhibit higher variability, reflecting a less consistent search behavior. This also suggests a less efficient balance between exploration and exploitation.

**Figure 5:**
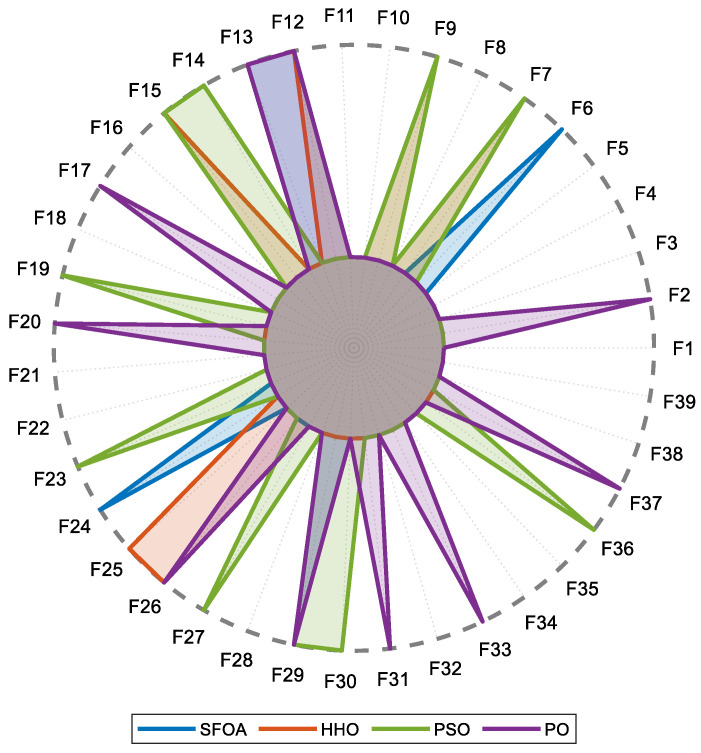
Radar plot illustrates the selected entropy-based features by SFOA, HHO, PSO, and PO.

The comparative feature selection behaviors of the optimization algorithms are further visualized using the radar plot presented in Figure 5. In addition, the index numbers of the extracted features and their corresponding entropy-based definitions are systematically listed in Table 3. All 39 features listed in Table 3 were directly extracted from the one-dimensional representations of tumor and non-tumor regions and constitute the complete entropy-based feature pool used for subsequent optimization and classification.

The results show that SFOA selects only five features (F6, F12, F13, F24, and F29), forming the most compact subset. In contrast, HHO selects six features (F7, F9, F12, F15, F25, and F26), PSO selects ten features (F7, F9, F14, F15, F19, F23, F27, F29, F30, and F36) and PO selects ten features (F2, F12, F13, F17, F20, F26, F29, F31, F33, and F37).

These findings suggest that SFOA is more effective at reducing dimensionality while maintaining classification performance. HHO provides a moderately inclusive subset, whereas PSO and PO adopt a broader, less selective feature selection strategy. Notably, the compact feature subset identified by SFOA has strong potential for generating parsimonious and generalizable representations in high-dimensional, entropy-based feature spaces. This aligns well with the principles of computational efficiency and model robustness.

The joint analysis of Table 2, Figure 4 and Figure 5 clearly demonstrates the effectiveness of the proposed SFOA-kNN feature selection strategy. Not only does SFOA achieve faster convergence and lower computational cost, but it also consistently identifies a more compact and informative feature subset than competing metaheuristic algorithms.

The integration of kNN within the wrapper-based optimization framework further amplifies this advantage. Since kNN relies on distance-based similarity, the elimination of redundant and noisy features directly enhances neighborhood consistency in the optimized feature space. As a result, the proposed SFOA-kNN configuration achieves a favorable balance between classification accuracy, feature compactness, and computational efficiency. These findings confirm that effective feature selection plays a critical role in improving classification performance and provide a strong foundation for the comparative classification analysis presented in the following subsection.

### 4.4. Evaluation and Discussion of Classification Models

The experimental analysis aims to investigate the influence of entropy-based features on the performance of both optimization and classification in discriminating between tumor and non-tumor brain regions. The proposed framework seeks to enhance classification accuracy while reducing computational complexity by systematically evaluating these factors.

The developed models were evaluated on a dataset consisting of 256 samples in total, including 128 tumor and 128 non-tumor regions. To assess model robustness and prevent biased evaluation, 10-fold cross-validation was employed for all classification experiments. The parameter settings of the classifiers used in this study are detailed in Table 4, ensuring reproducibility and fair comparison. As shown in Table 4, the kNN classifier was configured using the Euclidean distance metric with one nearest neighbor (k=1), whereas the SVM classifier employed a linear kernel with a box constraint value of 1. These configurations were selected to represent widely accepted and computationally efficient baseline settings for medical image classification tasks.

Since kNN is distance-based and sensitive to feature scaling, all entropy-based features were standardized prior to classification to ensure equal contribution in the Euclidean distance computation. The Euclidean metric was preferred due to the continuous and geometrically structured nature of the optimized entropy-based feature space.

The neighborhood size parameter was determined empirically by evaluating multiple k values, and k=1 achieved the highest classification performance. Given that the SFOA-optimized feature subset forms a compact and well-separated representation, smaller k values preserve sharper decision boundaries and enhance class discrimination.

For SVM, a linear kernel with c=1 was employed to provide balanced regularization while assessing the linear separability of the optimized feature space.

The performance of all classification models was evaluated using standard metrics derived from the confusion matrix, such as accuracy, precision, recall, and the F1-score. The quantitative results are summarized in Table 5. As can be seen in Table 5, the proposed SFOA-kNN model outperforms all other models across all evaluation metrics, achieving an overall classification accuracy of 99.20%. This confirms that the entropy-based features selected by SFOA are highly effective in discriminating between tumor and non-tumor regions.

Although the SFOA-SVM, HHO-kNN, PSO-kNN, and PO-kNN models also demonstrate high performance, none of them surpass the accuracy achieved by the proposed SFOA-kNN configuration. It is particularly noteworthy that SFOA-kNN attains its superior performance while utilizing only five entropy-based features, selected from the original set of 39 features, and within the shortest execution time reported in Table 2. This finding highlights the dual advantage of the proposed model in terms of both classification effectiveness and computational efficiency.

**Figure 6:**
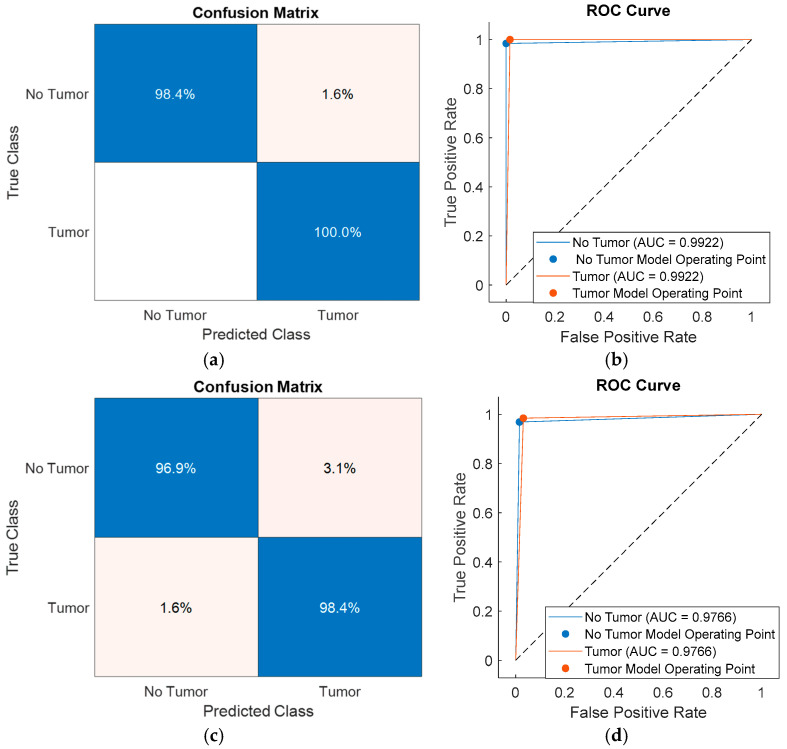
Confusion matrices and ROC curves of the proposed and comparative classification models: (**a**,**b**) SFOA-kNN and (**c**,**d**) SFOA-SVM.

**Figure 7:**
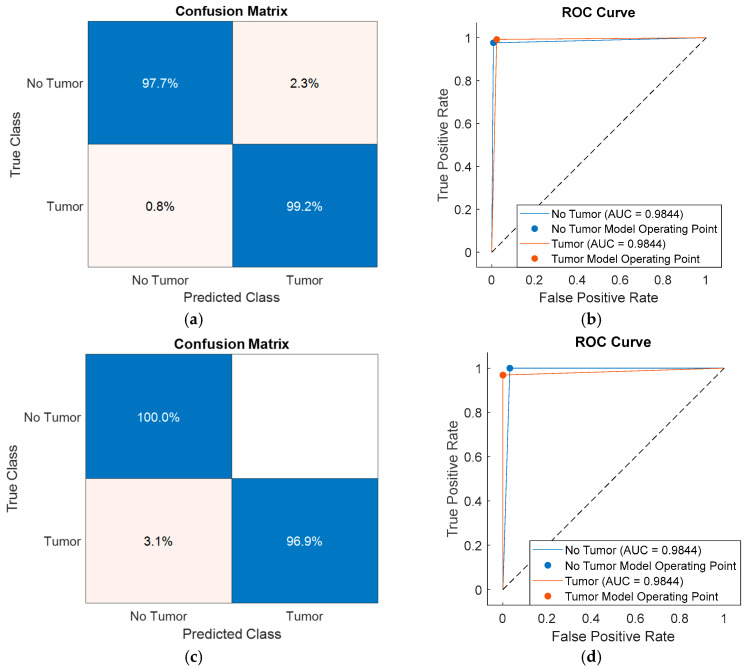
Confusion matrices and ROC curves of HHO-based classification models: (**a**,**b**) HHO-kNN and (**c**,**d**) HHO-SVM.

**Figure 8:**
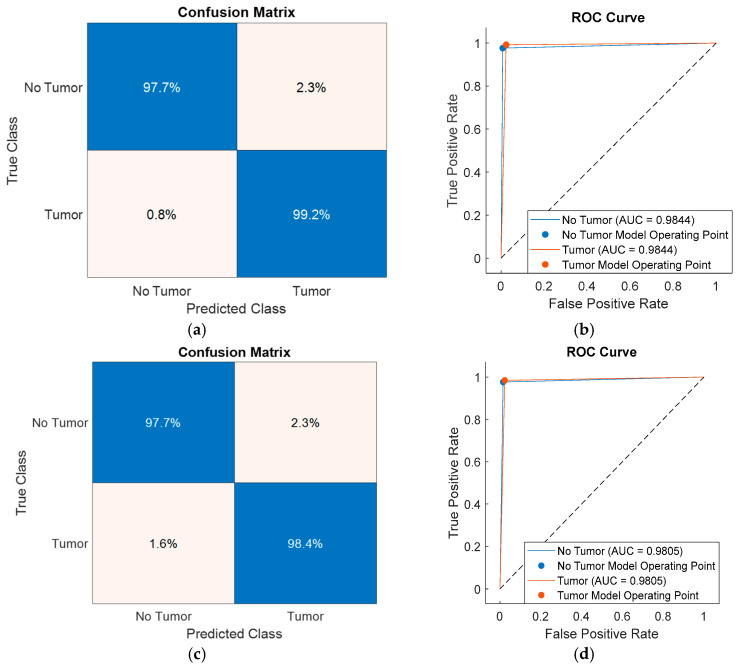
Confusion matrices and ROC curves of PSO-based classification models: (**a**,**b**) PSO-kNN and (**c**,**d**) PSO-SVM.

**Figure 9:**
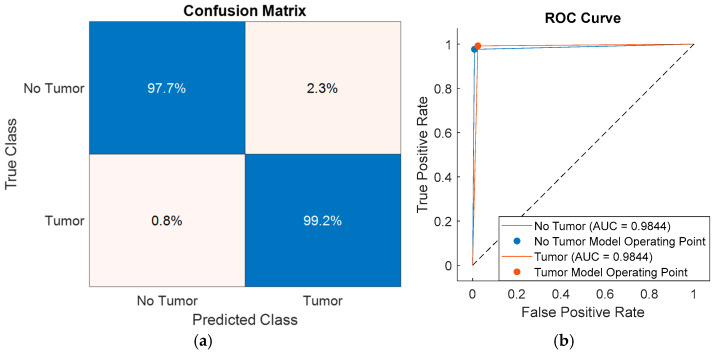

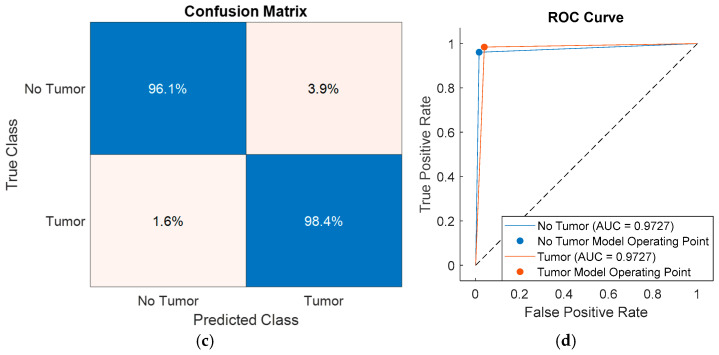
Confusion matrices and ROC curves of PO-based classification models: (**a**,**b**) PO-kNN and (**c**,**d**) PO-SVM.

To further analyze class-level performance, the classification results obtained using feature subsets selected by SFOA, HHO, PSO, and PO were visualized through confusion matrices and receiver operating characteristic (ROC) curves, as shown in Figure 6, Figure 7, Figure 8 and Figure 9. Figure 6 presents the performance of the SFOA-kNN and SFOA-SVM models, Figure 7 illustrates the results for the HHO-kNN and HHO-SVM models, Figure 8 depicts the corresponding outcomes for the PSO-kNN and PSO-SVM models, and Figure 9 shows the results for the PO-kNN and PO-SVM models.

The proposed SFOA-kNN model achieved 98.4% accuracy for non-tumor regions and 100% accuracy for tumor regions, indicating an almost perfect sensitivity to pathological tissue. In contrast, the SVM-based model obtained 96.9% accuracy for non-tumor regions and 98.4% accuracy for tumor regions. Although both classifiers achieved accuracy levels above 96% for all classes, the proposed SFOA-kNN model consistently outperformed the SVM-based model by approximately 1.6% for tumor classification and 1.5% for non-tumor classification.

These results suggest that the entropy features selected by SFOA are highly compatible with the local neighborhood-based decision structure of kNN, enabling clear separation between tumor and non-tumor samples. However, the same feature subsets do not contribute equally to the global, boundary-based decision mechanism of SVM, which limits the effectiveness of SVM-based models and reduces their reliability in this classification task.

The classification impact of feature subsets selected by HHO, PSO, and PO was also examined. Although HHO, PSO, and PO generated larger feature subsets consisting of six, ten, and ten features, respectively, these subsets failed to capture the most discriminative components as effectively as SFOA. The HHO-, PSO-, and PO-based models all achieved an area under the ROC curve (AUC) of around 0.972–0.984, indicating strong overall separability; however, they did not reach the class-level accuracy achieved by the proposed SFOA-kNN model.

Furthermore, the class separation in the HHO-, PSO-, and PO-based models was less pronounced in the confusion matrices and ROC curves than in the SFOA-kNN configuration. These findings suggest that selecting a larger number of features does not necessarily improve classification performance. Rather, the compact, highly discriminative subset of features identified by SFOA provides a more efficient, robust representation for entropy-based brain tumor classification.

The collective findings confirm that the feature subset selected by the optimization algorithm is a primary factor shaping classifier performance. Among all evaluated configurations, the proposed SFOA-kNN model achieves the best balance between low feature dimensionality, high classification accuracy, and minimal computational cost. While HHO, PSO, and PO also produce competitive results, they do not match the selectivity and efficiency achieved by SFOA. It is also noteworthy that SFOA strikes a good balance between low feature size, high classification accuracy and minimum computational cost compared to PO, a next-generation metaheuristic optimization algorithm. This confirms SFOA’s superiority to next-generation algorithms in entropy-based feature selection for brain tumor classification.

Considering the execution times reported in Table 2 alongside classification accuracy makes the superiority of the proposed model even more evident. Not only does the SFOA-kNN framework deliver the highest classification performance, but it also exhibits the lowest computational overhead, making it highly suitable for real-time and clinical decision support applications.

Overall, the experimental results demonstrate that the proposed approach can accurately classify tumor and non-tumor brain regions using a minimal number of entropy-based features. Thanks to its high accuracy, fast execution time, and low structural complexity, the proposed SFOA-kNN framework represents a reliable and clinically applicable solution for MRI-based brain tumor classification.

### 4.5. Comparative Analysis with Previous Studies Using the Same Dataset

To position the proposed framework within the existing literature, a comparative analysis was conducted with previous studies that utilized the same MRI dataset. Table 6 summarizes the methodological differences and reported performance metrics. Recent work by Pasunoori et al. proposed a hybrid YOLO11n + SAM framework for tumor detection and segmentation, achieving mAP@50 of 99.41%, precision of 98.1%, recall of 98.41%, Dice score of 96.06%, and IoU of 92.43%. Their approach focuses primarily on object detection and pixel-level segmentation performance [42]. Similarly, Gündoğdu and Akın conducted a comparative study between YOLOv8-seg and YOLOv11-seg models for tumor segmentation. Their results demonstrated high mAP@0.5 and mAP@0.5:0.95 performance values, emphasizing segmentation capability rather than post-detection classification [43].

In contrast, the present study introduces a fundamentally different pipeline. After YOLOv11-based localization, entropy-based feature extraction is performed on ROI regions, followed by SFOA-based wrapper optimization and classification via kNN. Therefore, unlike prior works that focus on detection or segmentation accuracy alone, the proposed framework evaluates the discriminative power of optimized entropy-based representations for tumor vs. non-tumor classification.

While segmentation-based studies report strong mAP and Dice scores, they do not analyze entropy-driven feature compactness, optimization efficiency, or classifier-level discrimination performance. The proposed SFOA-kNN framework achieves 99.20% classification accuracy using only five optimized entropy features, demonstrating superior compactness and computational efficiency compared to end-to-end segmentation pipelines.

These findings indicate that the proposed framework complements existing detection/segmentation approaches by introducing an optimized feature-based decision layer, thereby extending the analytical depth of MRI-based tumor assessment beyond spatial localization.

## 5. Conclusion and Future Research

This study presents an optimization-driven, hybrid machine learning framework for the accurate, computationally efficient classification of brain tumors using MRI. The proposed approach integrates YOLOv11-based automated tumor localization, entropy-driven feature extraction and metaheuristic feature subset optimization via SFOA, as well as final classification using a kNN classifier. Combining deep learning-based region detection with lightweight machine learning-based decision-making enables the framework to effectively address the challenges of high-dimensional feature redundancy, computational overhead and diagnostic reliability.

Extensive experimental evaluations demonstrated that the SFOA–kNN model consistently outperformed other optimization and classification strategies. Notably, an accuracy of 99.20% was achieved using only five entropy-based features, highlighting SFOA’s ability to identify compact, highly discriminative feature subsets. Comparative analyses against PSO-, HHO-, PO-, and SVM-based models further confirm that increasing model complexity or feature dimensionality does not necessarily translate into improved diagnostic performance, particularly when feature relevance is effectively optimized.

From a clinical perspective, the proposed framework offers a practical and scalable solution for computer-aided diagnostic systems. Automated tumor localization significantly reduces the need for manual intervention and variability between observers. Meanwhile, the compact feature representation enables rapid inference, making the framework suitable for real-time or near-real-time clinical workflows. Furthermore, using entropy-based features improves interpretability by providing transparent, quantitative descriptors that support clinical reasoning and boost the confidence among radiologists and other healthcare professionals.

Although the proposed framework demonstrates strong diagnostic performance and computational efficiency, the scope of the present study was intentionally focused on establishing a robust and well-controlled optimization-driven hybrid model. This design choice ensured that the core methodological contributions, automated tumor localization, entropy-based feature engineering, and metaheuristic feature selection, could be evaluated without introducing confounding variables that might obscure performance interpretation. Consequently, the current framework should be viewed as a validated and stable baseline rather than as an indication of methodological limitations.

From an implementation perspective, the current framework provides a solid foundation for future translational developments rather than highlighting methodological shortcomings. The binary classification setting adopted in this study is an important validation stage that ensures reliable tumor versus non-tumor discrimination before the model is extended to more specific diagnostic tasks. Based on this validated baseline, future research will naturally focus on multi-class tumor classification, including differentiation among clinically relevant tumor subtypes. This can be seamlessly integrated into the existing modular architecture without the need for structural modifications.

In addition, while the experimental evaluation was conducted using a well-curated, open-access dataset, the proposed framework is inherently agnostic to the dataset and is designed to accommodate diverse imaging protocols. Future work will therefore emphasize large-scale, multi-center validation and cross-institutional testing to facilitate clinical implementation and regulatory readiness. These evaluations will primarily serve as deployment-oriented extensions rather than methodological revisions, with the aim of demonstrating robustness across different scanners, acquisition settings and patient populations.

Another important area for future research is real-time system integration. Given the compact feature representation and low computational overhead achieved by the SFOA–kNN configuration, the framework is well positioned for deployment in clinical environments with limited computational resources. Future studies will focus on embedding the proposed pipeline into real-time computer-aided diagnosis platforms, evaluating system-level performance and latency, and assessing user interaction in routine clinical workflows. Furthermore, although entropy-based features are inherently interpretable, future implementations will explore integrating explainable artificial intelligence mechanisms to provide visual and quantitative explanations that align with radiological reasoning. This enhancement will further strengthen clinician trust and facilitate adoption in real-world diagnostic settings.

## Figures and Tables

**Table 1:** Parameter configurations of the metaheuristic optimization algorithms used for feature selection.

Parameter	SFOA	PSO	HHO	PO
Population size	50	50	50	50
Maximum iterations	100	100	100	100
Search space dimension	39	39	39	39
Lower bound	0	0	0	0
Upper bound	1	1	1	1
Fitness evaluation	kNN-based accuracy	kNN-based accuracy	kNN-based accuracy	kNN-based accuracy
Feature encoding	Binary	Binary	Binary	Binary
Inertia weight (ω)	-	0.9 → 0.4 (linear decrease)	-	-
Threshold	0.8	0.8	0.8	0.8
Cognitive coefficient (c1)	-	2.0	-	-
Social coefficient (c2)	-	2.0	-	-
Energy parameter (E)	-	-	Linearly decreasing	-
Randomization mechanism	Lévy-like movement	Uniform random	Adaptive random	Uniform random
Termination criterion	Max iterations	Max iterations	Max iterations	Max iterations

**Table 2:** Computational runtime and convergence performance of feature selection algorithms.

Optimization Algorithm	Total Running Time (s)
SFOA	0.8233
HHO	25.4099
PSO	16.9748
PO	10.8722

**Table 3:** Entropy-based features extracted in this study.

Feature ID	Feature Name
F1	Approximate Entropy (m=0)
F2	Approximate Entropy (m=1)
F3	Approximate Entropy (m=2)
F4	Attention Entropy
F5	Bubble Entropy
F6	Composite Multiscale Entropy (time scale 1)
F7	Composite Multiscale Entropy (time scale 2)
F8	Composite Multiscale Entropy (time scale 3)
F9	Shannon Entropy
F10	Corrected Conditional Entropy
F11	Cosine Similarity Entropy
F12	Dispersion Entropy
F13	Distribution Entropy
F14	Diversity Entropy
F15	Entropy of Entropy
F16	Fuzzy Entropy (m=1)
F17	Fuzzy Entropy (m=2)
F18	Gridded Distribution Entropy
F19	Permutation Entropy
F20	Phase Entropy
F21	Range Entropy
F22	Sample Entropy (m=0)
F23	Sample Entropy (m=1)
F24	Sample Entropy (m=2)
F25	Slope Entropy
F26	Spectral Entropy
F27	Cross-approximate Entropy (m=0)
F28	Cross-approximate Entropy (m=1)
F29	Cross-approximate Entropy (m=2)
F30	Corrected Cross-conditional Entropy
F31	Cross-distribution Entropy
F32	Cross-fuzzy Entropy (m=1)
F33	Cross-fuzzy Entropy (m=2)
F34	Cross-Kolmogorov Entropy (m=1)
F35	Cross-Kolmogorov Entropy (m=2)
F36	Cross-sample entropy (m=0)
F37	Cross-sample entropy (m=1)
F38	Cross-sample entropy (m=2)
F39	Cross-spectral Entropy

**Table 4:** Configuration parameters of kNN and SVM classifiers used in this study.

Classifier	Parameter	Value
kNN	Distance metric	Euclidean
	Number of neighbors	1
	Distance weighting	Uniform
	Feature standardization	Enabled
SVM	Kernel function	Linear
	Box constraint	1
	Kernel scale	Auto
	Feature standardization	Enabled

**Table 5:** Classification performance of the proposed and comparative models.

Model	Accuracy (%)	Precision (%)	Recall (%)	F1-Score (%)
SFOA-kNN	99.20	99.21	99.20	99.20
SFOA-SVM	97.65	97.66	97.65	97.65
HHO-kNN	98.45	98.46	98.45	98.45
HHO-SVM	98.45	98.49	98.45	98.45
PSO-kNN	98.45	98.46	98.45	98.45
PSO-SVM	98.05	98.05	98.05	98.05
PO-kNN	98.45	98.46	98.45	98.45
PO-SVM	97.25	97.27	97.25	97.26

**Table 6:** Comparative analysis of the proposed framework and previous studies conducted on the same MRI dataset.

Study	Task	Model	Feature Optimization	Accuracy/Map	Classification
[42]	Detection + Segmentation	YOLO11n + SAM	No	mAP@50: 99.41%	No
[43]	Segmentation	YOLOv8-seg/YOLOv11-seg	No	High mAP@0.5	No
Proposed Study	Detection + Feature-based Classification	YOLOv11 + SFOA + kNN	Yes (SFOA)	99.20% Accuracy	Yes

## Data Availability

The raw data supporting the conclusions of this article will be made available by the corresponding author on request.
